# Comparison of coated meshes for intraperitoneal placement in animal studies: a systematic review and meta-analysis

**DOI:** 10.1007/s10029-019-02071-y

**Published:** 2019-10-28

**Authors:** H. Liu, S. van Steensel, M. Gielen, T. Vercoulen, J. Melenhorst, B. Winkens, N. D. Bouvy

**Affiliations:** 1 Department of General Surgery, Maastricht University Medical Centre, PO Box 5800, 6202 AZ Maastricht, The Netherlands; 2 NUTRIM School of Nutrition and Translational Research in Metabolism, Maastricht University, Maastricht, The Netherlands; 3 Faculty of Health, Medicine and Life Sciences, Maastricht University, Maastricht, The Netherlands; 4 Department of Methodology and Statistics, CAPHRI, MUMC+, Maastricht, The Netherlands

**Keywords:** Surgical mesh, Tissue adhesion, IPOM, Systematic review, Meta-analysis

## Abstract

**Purpose:**

Laparoscopic intraperitoneal onlay mesh in hernia repair can result in adhesions leading to intestinal obstruction and fistulation. The aim of this systematic review is to compare the effects of mesh coatings reducing the tissue-to-mesh adhesion in animal studies.

**Methods:**

Pubmed and Embase were systematically searched. Animal experiments comparing intraperitoneally placed meshes with coatings were eligible for inclusion. Only studies with comparable follow-up, measurements, and species were included for data pooling and subsequent meta-analysis.

**Results:**

A total of 131 articles met inclusion criteria, with four studies integrated into one comparison and five studies integrated into another comparison. Compared to uncoated polypropylene (PP) mesh, PP mesh coated with hyaluronic acid/carboxymethyl cellulose (HA/CMC) showed significantly reduced adhesion formation at follow-up of 4 weeks measured with adhesion score of extent (random effects model, mean difference,−  0.96, 95% CI − 1.32 to − 0.61, *P* < 0.001, I^2^ = 23%; fixed effects model, mean difference,− 0.94, 95% CI − 1.25 to − 0.63, *P* < 0.001, *I*^2^ = 23%). Compared to PP mesh, polyester mesh coated with collagen (PC mesh) showed no significant difference at follow-up of 4 weeks regarding percentage of adhesion-area on a mesh, using random effects model (mean difference − 11.69, 95% CI − 44.14 to 20.76, *P* = 0.48, *I*^2^ = 92%). However, this result differed using fixed effects model (mean difference − 25.55, 95% CI − 33.70 to − 7.40, *P *< 0.001, *I*^2^ = 92%).

**Conclusion:**

HA/CMC coating reduces adhesion formation to PP mesh effectively at a follow-up of 4 weeks, while the anti-adhesive properties of PC mesh are inclusive comparing all study data.

**Electronic supplementary material:**

The online version of this article (10.1007/s10029-019-02071-y) contains supplementary material, which is available to authorized users.

## Introduction

Incisional hernia (IH) is one of the most common complications after laparotomy, with an incidence of around 13% after two years [1]. Generally, IH is defined as a gap or fascia defect, in the area of abdominal wall scars, that can be detected by clinical examination or imaging [2]. The number of patients having undergone IH repair was estimated to 3,00,000 in Europe in 2006 [3, 4]. Laparoscopic intraperitoneal onlay mesh placement (IPOM) is clinically available for hernia repair, usually indicated in patients with high risk of infection (diabetes, obesity, compromised immunity), recurrent hernia after open repair, swiss cheese hernia (multiple small defects), and lateral hernia (L1–L3) defects [5]. Meshes in IPOM position may result in adhesions leading to chronic pain [6], intestinal obstruction [7], difficulties at reoperation [8], and even fistulation [9, 10]. Consequently, protective layers were developed to coat the mesh in order to prevent adhesion formation.

Animal experiments are usually performed to test anti-adhesion effects of coatings applied to meshes, prior to potential translation to humans [11]. Although a large number of animal experiments have been conducted for this reason, systematic reviews of these comparisons are scarce [12]. It is a challenge to identify and compare meshes with identical conditions, i.e., identical species, follow-ups, measurements, and available data type. However, the number of animal experiments exploring an optimal coated mesh is increasing. A thorough overview of coatings to prevent adhesion formation on meshes is essential to the design of prospective animal experiments. The aim of this systematic review is to compare the effects of mesh coatings reducing the tissue-to-mesh adhesion in animal studies.

## Methods

This meta-analysis was performed according to the SYRCLE guidelines and registered at PROSPERO [nr: CRD42018089892].

### Inclusion and exclusion criteria

All animal studies investigating IH repair, comparing tissue-to-mesh adhesion intraperitoneally between a non-coated mesh and a coated mesh, or between coated meshes, were eligible for inclusion. Studies with identical type of two meshes (the same mesh material with the same coating material), comparable follow-up, identical measurements, and species were integrated in this meta-analysis.

Studies that only compared non-coated meshes or did not report data regarding tissue-to-mesh adhesions were excluded. Additionally, human trials, in vitro, and ex vivo experiments were excluded. There were no restrictions regarding species of animals, age, weight, and gender. Articles had to be written in English to be included. Studies were not excluded based on publication date.

### Search strategies

Pubmed and Embase were systematically searched on the 22nd of January, 2019. MeSH terms combined with free-text terms regarding IH repair, intraperitoneal mesh placement, and adhesion formation were used to search these databases. The full search strategies are available in table 1 and table 2 (Online Resource 1, ESM_1.PDF). The search was designed with the help of an experienced librarian from Maastricht University.

### Study selection

The search results were imported into a citation manager (EndNote™ X7, Clarivate Analytics). Duplicates were removed. After title and abstract screening, full-text screening was conducted by two independent researchers (MJ and TM) to identify the included articles. Disagreement was resolved by discussion and if needed a third researcher (HL) was contacted for arbitration.

### Data extraction

Data extraction was performed using a standard form, which included general study characteristics (the first author and the year of publication), animal characteristics (the animal species, the design of animal experiments, the animal model used, and the follow-up), mesh characteristics (types of the mesh, material and structures of the mesh, location of the placement, and fixation of the mesh), outcomes *(*extent score, tenacity score, adhesion-area, percentage of adhesion-area on a mesh, and adhesion incidence).

### Quality assessment

The quality of integrated studies were assessed by two independent researchers (HL and MJ), using the SYRCLE’s risk of bias tool [13]. This tool is an adapted version of the Cochrane risk of bias tool and specially developed for assessing the quality of animal studies. Briefly, this tool comprises ten items including the assessment of selection bias, performance bias, detection bias, attrition bias, reporting bias, and other bias.

### Data synthesis and statistical analysis

The meta-analysis was conducted using Review Manager (RevMan) [Computer program], Version 5.3 (Copenhagen: The Nordic Cochrane Centre, The Cochrane Collaboration, 2014). The identical comparisons of tissue-to-mesh adhesion at the same follow-up with the same species were pooled separately. In the comparison between polypropylene (PP) mesh and hyaluronic acid/carboxymethyl cellulose (HA/CMC) coated mesh, data of adhesion extent score were summarized to mean ± standard deviation if there were details reporting the extent score of adhesion or percentage of adhesion-area on mesh in every animal. The extent score of adhesion used the following score: grade 0, 0%; grade 1, 1–25%; grade 2, 26–50%; grade 3, 51–75%; and grade 4,   > 75% of mesh surface [14]. In the comparison between PP mesh and PC mesh, data of percentage of adhesion-area on mesh, presented by median, range and interquartile range, were conversed to mean ± standard deviation by formulas introduced by Wan X et al. [15] and principles in Cochrane handbook [16]. Percentage of adhesion-area was expressed as 100% × adhesion-area on the mesh/the mesh-area in the evaluation day. Treatment effects of data were expressed as mean difference with 95% confidence intervals and the inverse variance method was used. Both fixed effects model and random effects model were applied and *I*^2^ was used to express heterogeneity. If the pooled estimates are comparable between the fixed effects model and the random effects model, the results of the fixed effects model are preferred for the data integration. Otherwise, the results of the random effects model are preferred due to the obvious heterogeneity indicated by the results of two effects models [17].

## Results

**Fig. 1:**
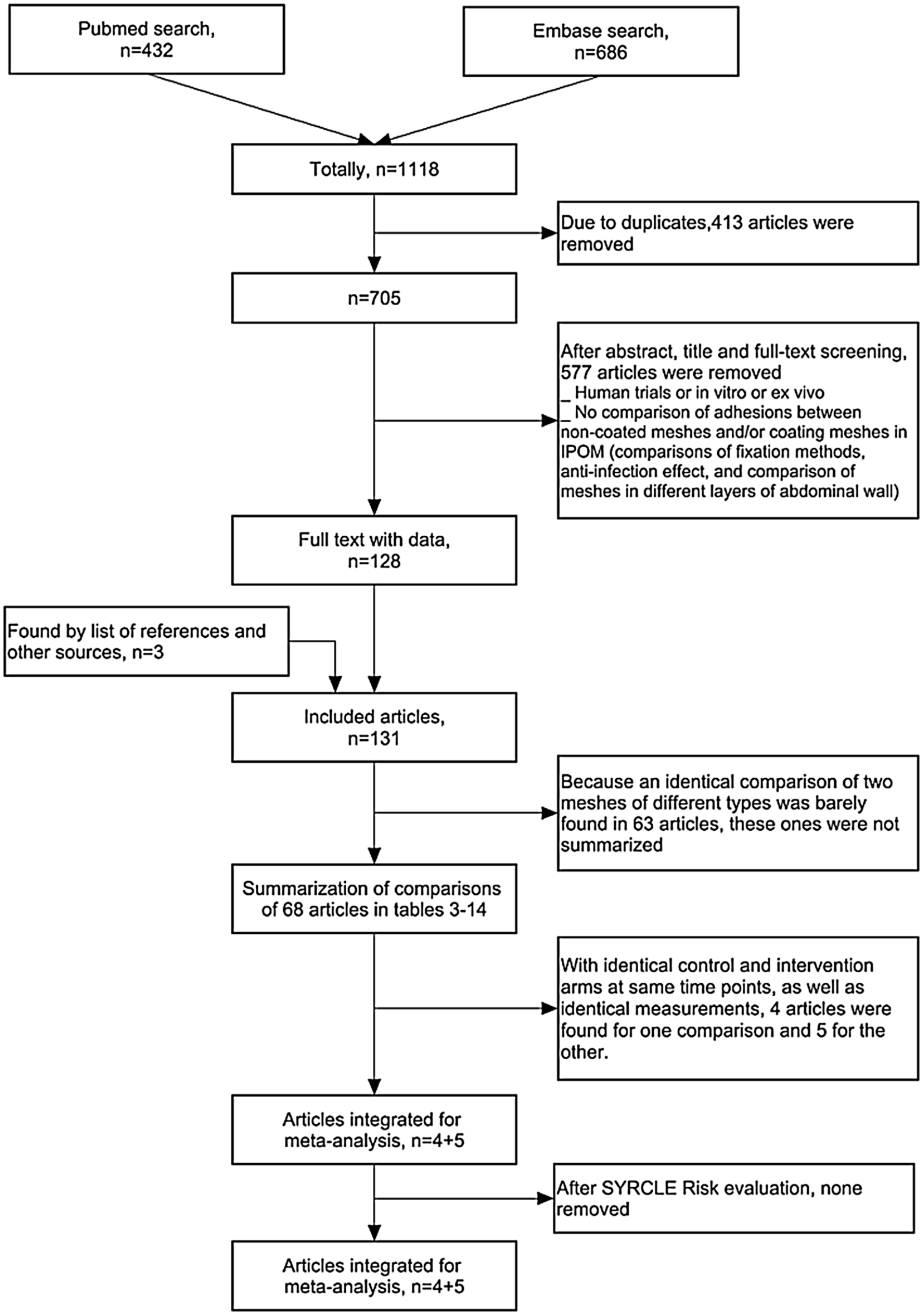
Flow diagram

A total of 705 articles were acquired from the search on Pubmed and Embase after removing the duplicates. The flow diagram (Fig. 1) displays the number of included, excluded and analyzed articles. Finally, 131 articles met the inclusion criteria. The comparisons of meshes usually applied in clinical practice were attached in Tables 3–14 (Online Resource 2, ESM_2.PDF), containing 68 articles. The other 63 articles were not summarized because an identical comparison of two meshes of different types was barely found in these articles. Four articles were pooled into meta-analysis for the comparison of PP mesh and HA/CMC mesh [18–21], and five articles for the comparison of PP mesh and PC mesh [22–26].

### Outcome of meta-analysis

#### Risk evaluation

**Fig. 2:**
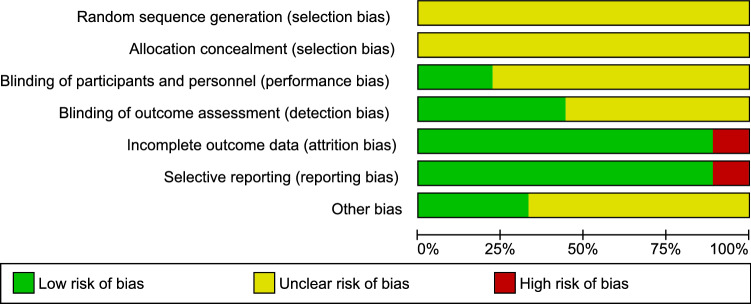
Risk of bias graph presented as a percentage of all included studies

**Fig. 3:**
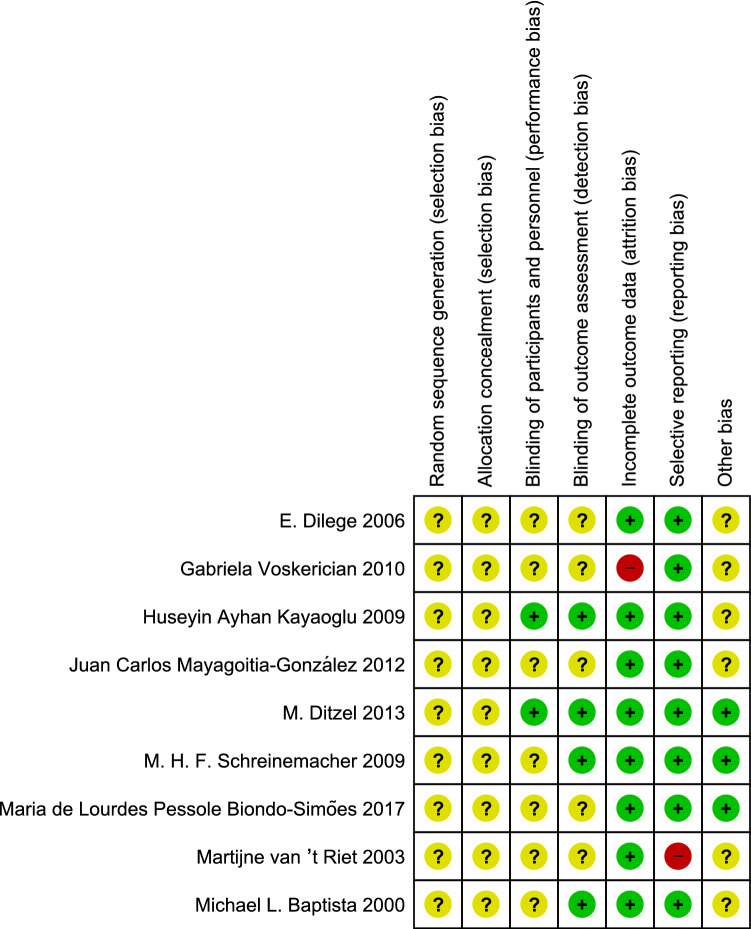
Risk of bias summary based on the judgement of reviews

All nine articles neither mentioned how random sequences were generated nor how allocation of animals was concealed [18–26] **(**Figs. 2, 3). Two out of these nine articles mentioned blinding of both performance and detection [20, 23], while two other articles mentioned only blinding of detection [24, 25]. One article was found with incomplete data, not mentioning the expression of data type [26], which implies that mean ± standard deviation or mean ± standard error cannot be identified directly from the article. One article was considered to show selective reporting [25] as animals without adhesion were not mentioned in their results.

#### Extent score of adhesion, PP mesh VS HA/CMC-coated PP mesh

**Fig. 4:**
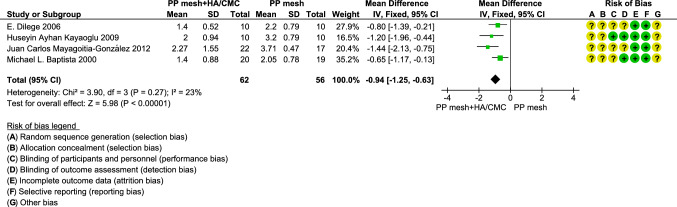
Comparison between polypropylene mesh and polypropylene mesh with HA/CMC coating in forest plot at 4 weeks follow-up in rats

Compared to PP mesh at a follow-up of 4 weeks in rats, HA/CMC-coated PP mesh demonstrated a significantly lower mean adhesion formation measured with extent score of adhesion (random effects model, mean difference, − 0.96, 95% CI − 1.32 to − 0.61, *P *< 0.001, *I*^2^ = 23%; fixed effects model, mean difference, – 0.94, 95% CI − 1.25 to –0 .63, *P* < 0.001, *I*^2^ = 23%). The results of the random effects and the fixed effects model in this comparison were similar, but the result of the fixed effects model was more precise due to the narrower confidence interval. Therefore, the result of the fixed effects model shown in Fig. 4 was preferred. In total, four studies with 118 animals were included in this comparison. The PP mesh in these four studies was Prolene® mesh (a heavy-weight PP mesh with medium pore size fabricated by monofilament) [19], Marlex® mesh (a heavy-weight PP mesh with medium pore size fabricated by monofilament) [18], Surgipro® mesh (a heavy-weight PP mesh with medium pore size fabricated by multifilament) [20], or a heavy polypropylene mesh [21]. The HA/CMC-coated PP mesh was Sepramesh® (PP mesh with HA/CMC coating) [19, 20], PP mesh covered by a HA/CMC membrane [18], or PP mesh coated by a HA/CMC gel [21]. The sutures for fixation of these meshes to abdominal wall were polypropylene sutures in three of four studies [18–20] and polyglyconate suture in one of four studies [21]. All these four studies showed HA/CMC-coated PP meshes were superior to PP meshes in adhesion reduction measured by extent score of adhesion.

#### Percentage of adhesion-area, PP mesh VS PC mesh

**Fig. 5:**
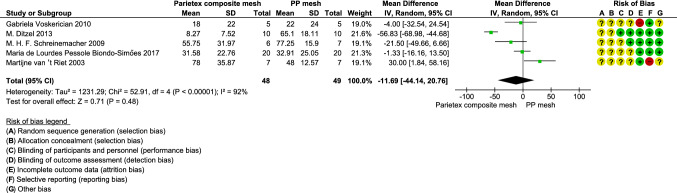
Comparison between polypropylene mesh and Parietex Composite mesh in forest plot at 4 weeks follow-up in rats

Compared to PP mesh at a follow-up of 4 weeks in rats, PC mesh showed a non-significant lower mean percentage of adhesion-area on mesh (random effects model, mean difference − 11.69, 95% CI − 44.14 to 20.76, *P* = 0.48, *I*^2^ = 92%; fixed effects model, mean difference –25.55, 95% CI − 33.70 to − 7.40, *P* < 0.001, *I*^2^ = 92%). The result of the random effects model shown in Fig. 5 was preferred for the integration, but the obvious heterogeneity was indicated by the results of two effects models. A total of five studies with 97 animals were included in this comparison. The PP mesh in four of five studies was Prolene® mesh [23–26], while Marlex® was used in the other study [22]. The PC mesh in all of these five studies was Parietex® Composite mesh. All the meshes in these five studies were secured to the abdominal wall with Prolene® sutures. Two of these five studies suggested PC mesh had less adhesion formation than PP mesh [23, 24], while two of five studies showed no benefit of PC mesh on adhesion reduction [22, 26] and one of five studies found PC mesh had higher adhesion formation than PP mesh [25].

## Discussion

Mesh use in IH significantly reduces recurrent IH compared with suture repair alone. However, an ideal mesh for intraperitoneal use remains to be explored due to mesh-related complications. Prosthetic meshes with novel coatings have been developed with high potential to overcome adhesion formation, one of the complications. While numerous animal studies have focused on the anti-adhesive effect of coated meshes, a systematic review investigating the optimal anti-adhesive mesh has not been conducted before. According to our results, HA/CMC-coated meshes and PC meshes were the most common meshes with coatings tested in animal experiments.

Seprafilm® membrane with a component of HA/CMC has been approved by FDA as a barrier for prevention of postoperative adhesion formation since 1996, with the support of several multi-center clinical trials [27, 28]. Due to technical difficulties caused by the adherent membrane used in IPOM, a new composite mesh was developed composed of PP mesh with a HA/CMC-coated layer facing the viscera. Most studies supported that HA/CMC-coated PP meshes were superior to PP mesh without a coating, although one study demonstrated no preference between both meshes [29]. Our meta-analysis results showed HA/CMC-coated PP meshes significantly reduced the adhesion formation measured by extent score at 4 weeks. Almost all animal studies included in this meta-analysis showed HA/CMC-coated PP mesh was preferred to PP mesh alone from 1 week follow-up to 4 months follow-up, in rats, rabbits, and pigs. The superiority of HA/CMC-coated PP meshes over uncoated PP mesh was found measured by percentage of adhesion-area on mesh, tenacity score of adhesion, extent score of adhesion, number of bowel adhesions to mesh, and rate of adhesion presence. Sasse et al. demonstrated that after HA/CMC-coated PP mesh (Sepramesh®, Davol, Providence, RI) was implanted intraperitoneally, little to no discomfort related to the mesh was reported in 65 of 72 (90.3%) patients over a follow-up period of 41 months [30]. Rose et al. provided a case illustrating that less than 25% of the HA/CMC-coated PP mesh (Sepramesh®, Genzyme, Biosurgery) was covered by filmy adhesion one year after the placement in patients [31]. Deeken et al. demonstrated to have initiated a multi-institutionally clinical trial focusing on the reductive effectiveness of coated meshes against adhesion [12].

PC mesh is another clinically available mesh with a layer of oxidized type 1 atelocollagen, polyethylene glycol, and glycerol coated on polyester prosthesis [32]. Some studies suggested that it significantly prevented adhesion formation, measured as percentage of adhesion-area, superior to PP mesh [20, 24, 29, 33–39]. However, some studies did not find a preference between PC mesh and PP mesh [22, 26]. One study even showed PC mesh presented higher adhesion formation than PP mesh [25]. Our meta-analysis showed no significant difference to be found between PC mesh and PP mesh in animal experiments using random effects model, noting the heterogeneity of these pooled studies was high. In humans, Chelala et al. reported that, detecting the PC mesh-related adhesions in patients with a second look operation, 40 of 85 (47.05%) patients were adhesion-free during a mean of 52-month follow-up using PC mesh, 36 of 85 (42.3%) were found to have loose adhesions to the omentum, and 9 of 85 (10.58%) patients had mild intestinal adhesions [40].

To our knowledge, this is the first time a meta-analysis was performed comparing coated meshes in the same conditions in animals. Although a large number of studies focused on evaluating tissue-to-mesh adhesions, animal studies with an identical comparison of meshes in the same conditions were scarce. This situation resulted from many different factors between groups, including mesh characteristics, measurement system for the adhesions, follow-up, and animal species.

To this moment, more than 70 types of meshes are commercially available [41]. Characteristics of a mesh, including coating, material absorption, pore size, weight, constitution, knitting structure, and fixation, played a role in the formation of tissue-to-mesh adhesions. Animal studies containing an identical comparison of two types of meshes, regarding the absolutely same characteristics of the two meshes, were scarce. The present two integrated studies only focused on the characteristics with the same mesh material and the same coating material.

The measurements of adhesion were generally classified based on percentage of adhesions covering a mesh, adhesion-area, incidence of adhesions, number of adhesions, type of adhesions, density of adhesions, and location of adhesions. No standard scoring system was widely accepted and applied in animal studies. Furthermore, even when aforementioned items were identical, grading of scores varies between studies, ranging from two levels to five levels. A consensus for researchers on a standard evaluation system of adhesion-to-mesh is urgently required.

Follow-up durations for evaluation of adhesion formation vary largely, from 3 days to one year in included studies, mainly due to differing research questions. Most follow-ups were set at 4 weeks. Generally, after in vivo implantation of biomaterials, multiple processes happen subsequently, including blood–material interaction, provisional matrix formation, acute and chronic inflammation, and finally formation of granulation tissue and fibrous encapsulation at 3–4 weeks [42]. Sulaiman et al. found that, in a mice adhesion model, peritoneal adhesion formation was mainly associated with chronic inflammation instead of acute inflammation [43]. Recommended by an expert consensus performed by our groups (unpublished), a follow-up of at least 4 weeks is suitable to assess chronic inflammation in animal experiments.

Appropriate animal species and models are necessary for animal studies. Especially since Van den Hil et al. found that histological outcomes were comparable between rats and humans, concerning adhesion formation and foreign body reaction to meshes [44].

Heterogeneity in meta-analysis represents between-studies variance, caused by different set-ups of the studies. To investigate heterogeneity of any significance, different statistical methods were applied after data integration of the pooled studies. Fixed effect models assume no heterogeneity exists and the variance between studies is fully caused by within-study variance. In contrast, random effects models include the possible effect of heterogeneity in pooled data [45]. In the current study, heterogeneity was identified in the studies comparing the PC mesh and PP mesh, despite mesh types, follow-up, species, and fixation material being virtually identical. It was not clear which other factors contribute to this heterogeneity. Subgroup meta-analysis detecting the heterogeneous source in the five pooled studies was complicated, due to the lack of details regarding randomization, animal housing conditions, the influence of different surgeons, and the microbiome, as well as unknown factors that might act as a source of the heterogeneity.

This meta-analysis encountered several limitations. Firstly, due to heterogeneity of all animal studies only several studies could be included in this meta-analysis and the sample size might not be large enough. This is due to the strict inclusion criteria and not enough studies available. The identical conditions for the comparisons were to ensure true integration of data. Secondly, the conversion of data might normally cause a little bias. Since all the data are converted under the same standards for the integration, consulting with the statistician in our university, bias seems minimal. Lastly, due to the different conditions between animals and humans, translation of our results to humans will still require human trials.

## Conclusion

HA/CMC coating reduces adhesion formation to PP mesh effectively at follow-up of 4 weeks, while the anti-adhesive properties of PC mesh are inclusive comparing all study data. A standard adhesion score assessing tissue-to-mesh adhesions is urgently required to reach a consensus for animal experiments.

## Electronic supplementary material

Below is the link to the electronic supplementary material.

Supplementary file1 (PDF 206 kb)

Supplementary file2 (PDF 485 kb)

## References

[1] Bosanquet DC, Ansell J, Abdelrahman T, Cornish J, Harries R, Stimpson A, Davies L, Glasbey JC, Frewer KA, Frewer NC, Russell D, Russell I, Torkington J (2015). Systematic review and meta-regression of factors affecting midline incisional hernia rates: analysis of 14,618 patients. PLoS ONE.

[2] Sanders DL, Kingsnorth AN (2012). The modern management of incisional hernias. BMJ.

[3] Sauerland S, Walgenbach M, Habermalz B, Seiler CM, Miserez M (2011). Laparoscopic versus open surgical techniques for ventral or incisional hernia repair. Cochrane Database Syst Rev.

[4] Poulose BK, Shelton J, Phillips S, Moore D, Nealon W, Penson D, Beck W, Holzman MD (2012). Epidemiology and cost of ventral hernia repair: making the case for hernia research. Hernia.

[5] Sharma A, Berger D (2018). The current role of laparoscopic IPOM repair in abdominal wall reconstruction. Hernia.

[6] Husain M, Sachan PK, Khan S, Lama L, Khan RN (2013). Role of diagnostic laparoscopy in chronic and recurrent abdominal pain. Trop Gastroenterol.

[7] Menzies D, Ellis H (1990). Intestinal obstruction from adhesions—How big is the problem?. Ann R Coll Surg Engl.

[8] ten Broek RP, Schreinemacher MH, Jilesen AP, Bouvy N, Bleichrodt RP, van Goor H (2012). Enterotomy risk in abdominal wall repair: a prospective study. Ann Surg.

[9] Burger JW, Luijendijk RW, Hop WC, Halm JA, Verdaasdonk EG, Jeekel J (2004). Long-term follow-up of a randomized controlled trial of suture versus mesh repair of incisional hernia. Ann Surg.

[10] Shubinets V, Carney MJ, Colen DL, Mirzabeigi MN, Weissler JM, Lanni MA, Braslow BM, Fischer JP, Kovach SJ (2018). Management of infected mesh after abdominal hernia repair: systematic review and single-institution experience. Ann Plast Surg.

[11] Hooijmans CR, Ritskes-Hoitinga M (2013). Progress in using systematic reviews of animal studies to improve translational research. PLoS Med.

[12] Deeken CR, Faucher KM, Matthews BD (2012). A review of the composition, characteristics, and effectiveness of barrier mesh prostheses utilized for laparoscopic ventral hernia repair. Surg Endosc.

[13] Hooijmans CR, Rovers MM, de Vries RB, Leenaars M, Ritskes-Hoitinga M, Langendam MW (2014). SYRCLE's risk of bias tool for animal studies. BMC Med Res Methodol.

[14] Diamond MP, Linsky CB, Cunningham T, Constantine B, diZerega GS, DeCherney AH (1987). A model for sidewall adhesions in the rabbit: reduction by an absorbable barrier. Microsurgery.

[15] Wan X, Wang W, Liu J, Tong T (2014). Estimating the sample mean and standard deviation from the sample size, median, range and/or interquartile range. BMC Med Res Methodol.

[16] Higgins JPT, Deeks JJ, Higgins JPT, Green S (2008). Selecting studies and collecting data Cochrane Handbook for Systematic Reviews of Interventions.

[17] Ryan R (2016) Cochrane Consumers and Communication Review Group. ‘Cochrane Consumers and Communication Group: meta-analysis‘. https://cccrg.cochrane.org. Accessed date December 2016.

[18] Baptista ML, Bonsack ME, Delaney JP (2000). Seprafilm reduces adhesions to polypropylene mesh. Surgery.

[19] Dilege E, Coskun H, Gunduz B, Sakiz D, Mihmanli M (2006). Prevention of adhesion to prosthetic mesh in incisional ventral hernias: comparison of different barriers in an experimental model. Eur Surg Res.

[20] Kayaoglu HA, Ozkan N, Hazinedaroglu SM, Ersoy OF, Erkek AB, Koseoglu RD (2005). Comparison of adhesive properties of five different prosthetic materials used in hernioplasty. J Investig Surg.

[21] Mayagoitia-Gonzalez JC, Gudino-Amezcua LM, Rivera-Barragan V, Mellado-Diaz AV, Diaz-Chavez EP (2012). Prevention of intestinal adhesions as a result of intraperitoneal mesh with the addition of hyaluronic acid/carboxymethylcellulose gel. Experimental model in rats. Cir Cir.

[22] Biondo-Simoes ML, Carvalho LB, Conceicao LT, Santos KB, Schiel WA, Arantes M, Silveira TD, Magri JC, Gomes FF (2017). Comparative study of polypropylene versus parietex composite(R), vicryl(R) and ultrapro(R) meshes, regarding the formation of intraperitoneal adhesions. Acta Cir Bras.

[23] Ditzel M, Deerenberg EB, Grotenhuis N, Harlaar JJ, Monkhorst K, Bastiaansen-Jenniskens YM, Jeekel J, Lange JF (2013). Biologic meshes are not superior to synthetic meshes in ventral hernia repair: an experimental study with long-term follow-up evaluation. Surg Endosc.

[24] Schreinemacher MH, Emans PJ, Gijbels MJ, Greve JW, Beets GL, Bouvy ND (2009). Degradation of mesh coatings and intraperitoneal adhesion formation in an experimental model. Br J Surg.

[25] van 't Riet M, van Steenwijk PJ, Bonthuis F, Marquet RL, Steyerberg EW, Jeekel J, Bonjer HJ (2003). Prevention of adhesion to prosthetic mesh: comparison of different barriers using an incisional hernia model. Ann Surg.

[26] Voskerician G, Jin J, White MF, Williams CP, Rosen MJ (2010). Effect of biomaterial design criteria on the performance of surgical meshes for abdominal hernia repair: a pre-clinical evaluation in a chronic rat model. J Mater Sci Mater Med.

[27] Becker JM, Dayton MT, Fazio VW, Beck DE, Stryker SJ, Wexner SD, Wolff BG, Roberts PL, Smith LE, Sweeney SA, Moore M (1996). Prevention of postoperative abdominal adhesions by a sodium hyaluronate-based bioresorbable membrane: a prospective, randomized, double-blind multicenter study. J Am Coll Surg.

[28] Beck DE, Cohen Z, Fleshman JW, Kaufman HS, van Goor H, Wolff BG, Adhesion Study Group Steering C (2003). A prospective, randomized, multicenter, controlled study of the safety of seprafilm adhesion barrier in abdominopelvic surgery of the intestine. Dis Colon Rectum.

[29] Gonzalez R, Rodeheaver GT, Moody DL, Foresman PA, Ramshaw BJ (2004). Resistance to adhesion formation: a comparative study of treated and untreated mesh products placed in the abdominal cavity. Hernia.

[30] Sasse KC, Lim DC, Brandt J (2012). Long-term durability and comfort of laparoscopic ventral hernia repair. JSLS.

[31] Rose J, Jayaraman S, Colquhoun P, Taylor B (2009). Minimal abdominal adhesions after Sepramesh repair of a parastomal hernia. Can J Surg.

[32] Briennon X, Lermite E, Meunier K, Desbois E, Hamy A, Arnaud JP (2011). Surgical treatment of large incisional hernias by intraperitoneal insertion of Parietex(R) composite mesh with an associated aponeurotic graft (280 cases). J Visc Surg.

[33] Schreinemacher MH, van Barneveld KW, Dikmans RE, Gijbels MJ, Greve JW, Bouvy ND (2013). Coated meshes for hernia repair provide comparable intraperitoneal adhesion prevention. Surg Endosc.

[34] LeBlanc KA, Bellanger D, Rhynes KVT, Baker DG, Stout RW (2002). Tissue attachment strength of prosthetic meshes used in ventral and incisional hernia repair. A study in the New Zealand white rabbit adhesion model. Surg Endosc.

[35] Gaertner WB, Bonsack ME, Delaney JP (2010). Visceral adhesions to hernia prostheses. Hernia.

[36] Jacob BP, Hogle NJ, Durak E, Kim T, Fowler DL (2007). Tissue ingrowth and bowel adhesion formation in an animal comparative study: polypropylene versus proceed versus parietex composite. Surg Endosc.

[37] Bellon JM, Garcia-Carranza A, Jurado F, Garcia-Honduvilla N, Carrera-San Martin A, Bujan J (2001). Peritoneal regeneration after implant of a composite prosthesis in the abdominal wall. World J Surg.

[38] Hu M, Lin X, Huang R, Yang K, Liang Y, Zhang X, Wang H, Wu D (2018). Lightweight, highly permeable, biocompatible, and antiadhesive composite meshes for intraperitoneal repairs. Macromol Biosci.

[39] Bellon JM, Rodriguez M, Garcia-Honduvilla N, Pascual G, Gil VG, Bujan J (2007). Peritoneal effects of prosthetic meshes used to repair abdominal wall defects: monitoring adhesions by sequential laparoscopy. J Laparoendosc Adv Surg Tech.

[40] Chelala E, Debardemaeker Y, Elias B, Charara F, Dessily M, Alle JL (2010). Eighty-five redo surgeries after 733 laparoscopic treatments for ventral and incisional hernia: adhesion and recurrence analysis. Hernia.

[41] Baylon K, Rodriguez-Camarillo P, Elias-Zuniga A, Diaz-Elizondo JA, Gilkerson R, Lozano K (2017). Past, present and future of surgical meshes: a review. Membranes (Basel).

[42] Sheikh Z, Brooks PJ, Barzilay O, Fine N, Glogauer M (2015). Macrophages, foreign body giant cells and their response to implantable biomaterials. Materials (Basel).

[43] Sulaiman H, Dawson L, Laurent GJ, Bellingan GJ, Herrick SE (2002). Role of plasminogen activators in peritoneal adhesion formation. Biochem Soc Trans.

[44] van den Hil LCL, Vogels RRM, van Barneveld KWY, Gijbels MJJ, Peutz-Kootstra CJ, Cleutjens JPM, Schreinemacher MHF, Bouvy ND (2018). Comparability of histological outcomes in rats and humans in a hernia model. J Surg Res.

[45] Barili F, Parolari A, Kappetein PA, Freemantle N (2018). Statistical primer: heterogeneity, random- or fixed-effects model analyses?. Interact Cardiovasc Thorac Surg.

