# Supplementary material for: Comparison of coated meshes for intraperitoneal placement in animal studies: a systematic review and meta-analysis
Source: Hernia. 2019 Oct 28;24(6):1253–61. doi: 10.1007/s10029-019-02071-y (PMC7701080; doi:10.1007/s10029-019-02071-y)
Supplement: Supplementary file 2 — Supplementary file2 (PDF 485 kb) [file 10029_2019_2071_MOESM2_ESM.pdf]

Article Title:

Comparison of coated meshes for intraperitoneal placement in animal studies, a systematic review and meta-analysis

Journal name:

Hernia: the journal of hernias and abdominal wall surgery

Authors:

Hong Liu, Sebastiaan van Steensel, Maurits-Jan Gielen, Timon Vercoulen, Jarno Melenhorst, Bjorn Winkens, Nicole D. Bouvy

Correspondence:

Address: Hong Liu

Department of General Surgery, Maastricht University Medical Centre

PO Box 5800, 6202 AZ, Maastricht, the Netherlands

E-mail: h.liu@maastrichtuniversity.nl

Table 3 The comparison between polypropylene mesh and HA/CMC coated PP mesh (PP mesh VS PP mesh + HA/CMC)

Species	Follow-up	Comparison (PP mesh VS PP mesh + HA/CMC)	Best mesh	Measurements and reference
Rat	Several time points together till 4 weeks	Surgipromesh (AutoSuture) VS PP mesh + CMC [1];<br>PP mesh VS Sepramesh (Genzyme) [2]	PP mesh + HA/CMC	Extent score of adhesion [1];<br>Rate of adhesion presence [2];<br>Percentage of adhesion-area [2]
Rat	1 week	Prolene (Ethicon) VS PP mesh + HA [3];<br>Prolene (Ethicon) VS Sepramesh (Genzyme) [4,5];<br>Marlex (Bard) VS PP mesh + Seprafilm (Genzyme) [6]	PP mesh + HA/CMC	Tenacity score of adhesion [3];<br>Numbers of rats with bowel adhesion [5];<br>Percentage of adhesion-area [5,4,6]
Rat<br>Rabbits	2 weeks	PP mesh VS PP mesh + Seprafilm (Genzyme) [7];<br>PP mesh VS PP mesh + Sepracoat (Genzyme) [8];<br>Marlex (Bard) VS Sepramesh (Genzyme) [9];<br>Marlex(Bard) VS PP mesh + Seprafilm (Genzyme) [6]	PP mesh + HA/CMC	Percentage of adhesion-area [7] (rat), [8,9] (rabbit), [6] (rat);<br>Adhesion score composed of type and tenacity [9] (rabbit)
Rat	3 weeks	PP mesh VS PP mesh + Seprafilm (Genzyme)	PP mesh + HA/CMC	Percentage of adhesion-area [10]
Rat	3 weeks	Parietene (Sofradim) VS Sepramesh (Genzyme)	No preference	Adhesion-area [11];<br>Rate of adhesion presence [11]
Rat<br>Rabbit<br>Pig	4 weeks	PP mesh VS Sepramesh (Genzyme) [12]<br>Prolene(Ethicon) VS Sepramesh (Genzyme) [4,5];<br>Marlex (Bard) VS Seprafilm (Genzyme) [6];<br>Heavy PP mesh VS PP mesh + HA/CMC gel [13];<br>PP mesh (Davol) VS Sepramesh (Genzyme) [14];<br>PP mesh VS PP mesh + Seprafilm (Genzyme) [15];<br>Prolene (Ethicon) VS Sepramesh (Genzyme) [16,17];<br>Surgipro (AutoSuture) VS Sepramesh (Genzyme) [18]	PP mesh + HA/CMC	Percentage of adhesion-area [12] (pig), [4,6,5,13] (rat), [14,15] (rabbit);<br>Tenacity score of adhesion [16-18] (rat);<br>Extent score of adhesion [17,18,13] (rat), [14] (rabbit);<br>Severity score of adhesion [14] (rabbit), [13] (rat);<br>Density score of adhesion [13] (rat);<br>Rate of adhesion presence [14] (rabbit);<br>Adhesion numbers from organs to mesh [15] (rabbit)
Rat	4 weeks	Prolene (Ethicon) VS PP mesh + SCMC	PP mesh + SCMC	Extent score of adhesion [19]
Rat<br>Pig	6 weeks	Surgipro VS Seprafilm (Genzyme) [20];<br>Premilene(Braun) VS PP mesh + Seprafilm (Genzyme) [21];<br>PP mesh VS PP mesh + Seprafilm (Genzyme) [10]	PP mesh + HA/CMC	Percentage of adhesion-area [20] (pig), [10] (rat);<br>Rate of adhesion presence [21] (rat)
Rat	8 weeks	Prolene (Ethicon) VS Sepramesh (Genzyme) [22];<br>PP mesh VS Seprafilm (Genzyme) [23]	PP mesh + HA/CMC	Percentage of adhesion-area [22];<br>Adhesion score composed of type and tenacity [22];<br>Adhesion score composed of extent and tenacity [23];<br>Number of rats with severity score of adhesion 3-4 [22]
Rat	3 months	Parietene (Covidien) VS Sepramesh (Bard)	PP mesh + HA/CMC	Percentage of adhesion-area [24];<br>Adhesion score composed of extent, type and tenacity [24]
Rabbit	4 months	ProLite Ultra mesh VS Sepramesh (Bard)	PP mesh + HA/CMC	Percentage of adhesion-area [25];<br>Tenacity score of adhesion [25]

Table 4 The comparison between PP mesh and Parietex Composite mesh (PP mesh VS Polyester mesh + collagen)

Species	Follow-up	Comparison<br>(PP mesh VS Polyester mesh + collagen)	Best mesh	Measurements and reference
Rat	1 week	Prolene (Ethicon) VS Parietex Composite (Sofradim)	No preference	Percentage of adhesion-area [5]
Rat	1 week	Prolene (Ethicon) VS Parietex Composite (Sofradim) [4,26,5]	Polyester mesh + collagen	Numbers of rats with bowel adhesion [5];<br>Percentage of adhesion-area [4,26];<br>Adhesion score composed of extent, type and tenacity [26]
Rabbit	2 weeks	Surgipro (Tyco) VS Parietex Composite (Sofradim) [27] ;<br>Prolene (Ethicon) VS Parietex Composite (Sofradim) [28]	Polyester mesh + collagen	Percentage of adhesion-area [27];<br>Adhesion-area [28]
Rat	3 weeks	Parietene (Sofradim) VS Parietex Composite (Sofradim) [11];<br>Marlex VS Parietex Composite [29]	Polyester mesh + collagen	Percentage of adhesion-area [11,29];<br>Rate of adhesion presence [11];<br>Tensile strength of adhesion [29]
Rat	4 weeks	Prolene (Ethicon) VS Parietex Composite [5]	PP mesh	Percentage of adhesion-area [5]
Rat	4 weeks	Marlex VS Parietex Composite [30];<br>Prolene (Ethicon) VS Parietex Composite (Covidien) [31]	No preference	Percentage of adhesion-area [30,31];<br>Extent score of adhesion [31];<br>Tenacity score of adhesion [31]
Rat	4 weeks	Prolene (Ethicon) VS Parietex Composite (Sofradim) [4,26];<br>Prolene (Ethicon) VS Parietex Composite (Covidien) [32];<br>Surgipro (AutoSuture) VS Parietex Composite (Sofradim) [18]	Polyester mesh + collagen	Percentage of adhesion-area [4,32,26];<br>Extent score of adhesion [18];<br>Tenacity score of adhesion [18];<br>Adhesion score composed of extent, type and tenacity [26];<br>Adhesion score composed of tenacity and severity [32]
Rat	3 months	Prolene (Ethicon) VS Parietex Composite (Covidien) [32];<br>Parietene (Covidien) VS Parietex Composite (Covidien) [24]	Polyester mesh + collagen	Adhesion score composed of tenacity and severity [32];<br>Percentage of adhesion-area [32];<br>Adhesion score composed of extent, type and tenacity [24]
Rabbit	4 months	ProLite Ultra VS Parietex Composite (Covidien)	No preference	Tenacity score of adhesion [25];<br>Percentage of adhesion-area [25]

Table 5 The comparison between PP mesh and Parietene Composite mesh (PP mesh VS PP mesh + collagen)

Species	Follow-up	Comparison<br>(PP mesh VS PP mesh + collagen)	Best mesh	Measurements and reference
Rat	1 week	Prolene (Ethicon) VS coated Parieten (Sofradim)	No preference	Rate of adhesion presence [33];<br>Number of rats with bowel adhesion [33]
Rat	3 weeks	Parietene (Sofradim) VS Parietene Composite (Sofradim)	PP mesh + collagen	Rate of adhesion presence [11];<br>Percentage of adhesion-area [11]
Rat	4 weeks	Prolene (Ethicon) VS coated Parieten (Sofradim)	PP mesh + collagen	Rate of adhesion presence [33]
Rat	4 weeks	Prolene (Ethicon) VS coated Parieten (Sofradim)	No preference	Number of rats with bowel adhesion [33]
Rat	2 months	Prolene (Ethicon) VS Parietene Composite (Sofradim)	No preference	Percentage of adhesion-area [22];<br>Severity score of adhesion [22]
Rat	2 months	Prolene (Ethicon) VS Parietene Composite (Sofradim)	PP mesh + collagen	Number of rats with severity score of adhesion 3-4 [22]
Rat	3 months	Parietene (Covidien) VS Parietene Composite (Covidien)	PP mesh + collagen	Percentage of adhesion-area [24];<br>Adhesion score composed of extent, type and tenacity [24]

Table 6 The comparison between PP mesh and PP mesh coated by polyurethane (PP mesh VS PP mesh + PU)

Species	Follow-up	Comparison<br>(PP mesh VS PP mesh + PU)	Best mesh	Measurements and reference
Rat	2 weeks	Marlex (Bard) VS PP mesh + PU	No preference	Percentage of adhesion-area [34]
Rabbit	2 weeks	PP mesh VS PP mesh + PU	PP mesh + PU	Adhesion-area [35]
Rat<br>Rabbit	2 weeks	Marlex (Bard) VS PP mesh + PU [34];<br>Surgipro (Tyco) VS PP mesh + PU [27]	PP mesh + PU	Severity score of adhesion (≥moderate) [34];<br>Percentage of adhesion-area [27]
Rat	3 weeks,<br>3 months,<br>6 months<br>together	Prolene (Ethicon) VS Combi (Angiologica)	PP mesh + PU	Extent score of adhesion [36];<br>Tenacity score of adhesion [36]

Table 7 The comparison between PP mesh and PP-polydioxanone mesh coated by oxidized regenerated cellulose (PP mesh VS PP mesh + ORC)

Species	Follow-up	Comparison<br>(PP mesh VS PP mesh + ORC)	Best mesh	Measurements and reference
Rat	1 week,<br>4 weeks<br>together	Surgipromesh (AutoSuture) VS PP mesh + Interceed TC7 (Johnson & Johnson)	PP mesh + ORC	Extent score of adhesion [1]
Rat	1 week	Prolene (Ethicon) VS coated Proceed (Ethicon) [37,26]	PP mesh + ORC	Percentage of adhesion-area [26];<br>Adhesion score composed of extent, type and tenacity [26,37]
Rat	1 week	Prolene (Ethicon) VS coated Proceed (Ethicon)	No preference	Percentage of adhesion-area [4]
Rat	2 weeks	Bard Mesh (Bard) VS PP mesh + Interceed TC7 (Ethicon)	PP mesh + ORC	Scale score of adhesion [38];<br>Percentage of adhesion–area [38]
Rat	4 weeks	Prolene (Ethicon) VS Proceed (Ethicon) [4,37,26]	No preference	Percentage of adhesion-area [4,26];<br>Tenacity and extent score of adhesion [37];<br>Adhesion score composed of extent, type and tenacity [26]
Rat	4 weeks	Prolene (Ethicon) VS PP mesh + Interceed (Johnson & Johnson)	PP mesh + ORC	Extent score of adhesion [17];<br>Tenacity score of adhesion [17]
Pig	6 weeks	Prolene (Ethicon) VS PP mesh + Interceed (Johnson & Johnson)	PP mesh + ORC	Percentage of adhesion-area [39]
Pig	6 weeks	Prolene (Ethicon) VS PP mesh + Interceed (Johnson & Johnson)	No preference	Percentage of adhesion-area in laparoscopy [39];<br>Tenacity score of adhesion in laparoscopy [39]
Rabbit	8 weeks	PREMILENE (B. Braun) VS Proceed (Johnson & Johnson)	PP mesh + ORC	Extent score of adhesion [40]
Rabbit	8 weeks	PREMILENE (B. Braun) VS Proceed (Johnson & Johnson)	No preference	Adhesion size [40];<br>Adhesion separate strength [40];<br>Adhesion score composed of extent, size and strength to separate from mesh [40]
Rat	3 months	Marlex (Bard) VS Proceed (Ethicon)	No preference	Adhesion score composed of extent and tenacity [41]
Rabbit	4 months	ProLite Ultra mesh VS Proceed (Ethicon)	No preference	Adhesion score of tenacity [25];<br>Percentage of adhesion-area [25]
Pig	7 months	Prolene (Ethicon) VS Proceed (Ethicon)	No preference	Percentage of adhesion-area in laparoscopy [39];<br>Tenacity score of adhesion in laparoscopy [39]
Rabbit	1 year	Marlex (Bard) VS Proceed (Ethicon)	PP mesh + ORC	Percentage of adhesion-area [42]

Table 8 The comparison between PP mesh and polyglactin coated PP mesh (PP mesh VS PP mesh + polyglactin)

Species	Follow-up	Comparison (PP mesh VS PP mesh + polyglactin)	Best mesh	Measurements and reference
Rabbit	2 weeks	Marlex(Bard) VS Marlex (Bard) + Vicryl (Ethicon)	PP mesh + polyglactin	Percentage of adhesion-area [9];<br>Adhesion score composed of type and tenacity [9]
Rat	2 weeks	Parietene (Tyco) VS VyproII (Ethicon);<br>Optilene (B/Braun) VS VyproII (Ethicon)	No preference	Percentage of adhesion-area [43]
Rat<br>Rabbit	4 weeks	Prolene (Ethicon) VS Prolene (Ethicon) + Vicryl (Ethicon) [44];<br>Prolene (Ethicon) VS VyproII (Ethicon) [45]	No preference	Tenacity score of adhesion [44] (rat);<br>Rate of adhesion presence [45] (rabbit);<br>Adhesion score of tenacity and type [45] (rabbit)
Rat	4 weeks	Surgipro mesh (AutoSuture) VS Vypro II (Johnson & Johnson)	PP mesh + polyglactin	Extent score of adhesion [18];<br>Tenacity score of adhesion [18];<br>Breaking force of adhesion [18]
Rat<br>Pig	2 months	Prolene (Ethicon) VS Prolene (Ethicon) + Vicryl (Ethicon) [44];<br>Marlex (Bard) VS Marlex (Bard) + Vicryl (Ethicon) [46];<br>Softmesh (Bard) VS Softmesh (Bard) + Vicryl (Ethicon) [46]	No preference	Extent score of adhesion [46] (pig);<br>Tenacity score of adhesion [44] (rat), [46] (pig)
Rabbit	3 months	PP mesh VS PP mesh + polyglactin	PP mesh + polyglactin	Percentage of adhesion-area [43]

Table 9 The comparison between PP mesh and PP mesh covered by expanded polytetrafluoroethylene (PP mesh VS PP mesh + ePTFE)

Species	Follow-up	Comparison<br>(PP mesh VS PP mesh +ePTFE)	Best mesh	Measurements and reference
Rat	3 weeks, 3 months,<br>6 months together	Prolene (Ethicon) VS Bard Composix (Bard)	PP mesh + ePTFE	Extent score of adhesion [36];<br>Tenacity score of adhesion [36]
Rat<br>Rabbit	2 weeks	Surgipro (Tyco) VS Bard Composix (Bard) [27];<br>Prolene (Marlex) VS Bard Composix (Bard) [7];<br>Bard Mesh (Bard) VS Bard Composix (Bard) [38];<br>Prolene (Ethicon) VS Prolene (Ethicon) + ePTFE (Gore-Tex) [28]	PP mesh + ePTFE	Percentage of adhesion-area [7] (rat), [27] (rabbit), [38] (rat);<br>Scale score of adhesion [38] (rat);<br>Adhesion-area [28] (rabbit)
Rat	3 weeks	Parietene (Sofradim) VS Bard Composix E/X (Bard)	PP mesh + ePTFE	Rate of adhesion presence [11];<br>Percentage of adhesion-area [11]
Rabbit	4 weeks	PP mesh (Davol) VS Bard Composix (Davol)	PP mesh + ePTFE	Extent score of adhesion [14]
Rabbit	4 weeks	PP mesh (Davol) VS Bard Composix (Davol)	No preference	Percentage of adhesion-area [14];<br>Adhesion presence rate [14]
Rat	2 months	Prolene (Ethicon) VS Bard Composix (Bard)	No preference	Percentage of adhesion-area [22];<br>Adhesion score composed of type and tenacity [22]
Rat	2 months	Prolene (Ethicon) VS Bard Composix (Bard)	PP mesh + ePTFE	Number of rats with adhesion score 3-4 [22];
Rabbit	120 days	ProLite Ultra VS Bard Composix (Bard)	No preference	Tenacity score of adhesion [25];<br>Percentage of adhesion-area [25]
Rabbit	1 year	PP mesh (Marlex) VS Bard Composix (Bard)	PP mesh + ePTFE	Percentage of adhesion-area [42]

Table 10 The comparison between PP mesh and C-Qur mesh (PP mesh VS C-QUR mesh)

Species	Follow-up	Comparison<br>(PP mesh VS C-QUR mesh)	Best mesh	Measurements and reference
Rat	7 days	Prolene (Ethicon) VS C-Qur (Atrium)	C-Qur mesh	Percentage of adhesion-area [26];<br>Adhesion score composed of extent, type and tenacity [26]
Rat	30 days	Prolene (Ethicon) VS C-Qur (Atrium)	C-Qur mesh	Percentage of adhesion-area [26];<br>Adhesion score composed of extent, type and tenacity [26]
Rat	90 days	Parietene (Covidien) VS C-Qur (Atrium)	C-Qur mesh	Percentage of adhesion-area [24];<br>Adhesion score composed of extent, type and tenacity [24]
Rabbit	120 days	ProLite Ultra VS C-Qur (Atrium)	No preference	Tenacity score of adhesion [25];<br>Percentage of adhesion-area [25]

Table 11 The comparison between PP mesh coated by collagen (Parietene Composite mesh) VS other meshes with coatings

Species	Follow-up	Comparison	Best mesh	Measurements and reference
Rat	3 weeks	Parietene (Sofradim) VS Parietex Composite (Sofradim)	PP mesh + collagen	Rate of adhesion presence [11];<br>Percentage of adhesion-area [11]
Rat	3 weeks	Parietene Composite (Sofradim) VS Parietex Composite (Sofradim)<br>Parietene Composite (Sofradim) VS Bard Composix E/X (Davol)	No preference	Rate of adhesion presence [11];<br>Percentage of adhesion-area [11]
Rat	2 months	Parietene Composite (Sofradim) VS PP mesh + Hyalobarrier gel<br>Parietene Composite (Sofradim) VS PP mesh + Tissucol (Baxter Healthcare)<br>Parietene Composite (Sofradim) VS Bard Composix (Bard)<br>Parietene Composite (Sofradim) VS Sepramesh (Genzyme)	No preference	Percentage of adhesion-area [22];<br>Adhesion score composed of type and tenacity [22]
Rat	3 months	Parietene Composite (Covidien) VS Parietex Composite (Covidien)<br>Parietene Composite (Covidien) VS C-Qur (Atrium)<br>Parietene Composite (Covidien) VS Sepramesh IP (Bard)	No preference	Percentage of adhesion-area [24];<br>Adhesion score composed of extent, type and tenacity [24]
Pig	4 months	Parietene Composite (Covidien) VS Dynamesh IPOM (GmbH);<br>Parietene Composite (Covidien) VS Proceed (Ethicon)	PP mesh + collagen	Percentage of adhesion-area [47]

Table 12 The comparison between PP mesh coated by HA/CMC (Sepramesh) and other kinds of meshes with coatings

Species	Follow-up	Comparison	Best mesh	Measurements and reference
Rat	1 week, 4 weeks together	PP mesh + Interceed TC7 (Johnson & Johnson) VS PP mesh + CMC	No preference	Extent score of adhesion [1]
Rabbit	3 days	Sepramesh (Genzyme) VS Parietex Composite (Sofradim)<br>Sepramesh (Genzyme) VS PP mesh + polyurethane	PP mesh + HA/CMC	Percentage of mesh adhesion-area [48]
Rabbit	3 days	Sepramesh (Bard) VS Parietex Composite (Covidien)	Polyester mesh + collagen	Rate of adhesion presence [49]
Rabbit	3 days	Sepramesh (Bard) VS Proceed (Ethicon)	No preference	Rate of adhesion presence [49]
Rat<br>Rabbit	1 week	Sepramesh (Bard) VS Parietex Composite (Covidien) [50];<br>Sepramesh (Genzyme) VS Parietex Composite (Sofradim) [5];<br>Sepramesh (Bard) VS Physiomesh (Ethicon) [50];<br>Sepramesh (Genzyme) VS DualMesh (GORE-TEX) [51];<br>Sepramesh (Bard) VS Proceed (Ethicon) [49]	No preference	Percentage of adhesion-area [5] (rat);<br>Numbers of rats with bowel adhesion [5];<br>Adhesion score composed of width, thickness, subjective strength and amount of adhesion [50] (rabbit);<br>Extent score of adhesion [51] (rabbit);<br>Rate of adhesion presence [49] (rabbit)
Rat<br>Rabbit	1 week	Sepramesh (Genzyme) VS Ultrapro (Ethicon) [4];<br>Sepramesh (Genzyme) VS Timesh (GfE Medizintechnik GmbH) [4];<br>Sepramesh (Bard) VS Bard Composix (Bard) [50];<br>Sepramesh (Bard) VS Proceed (Johnson & Johnson) [50];<br>Sepramesh (Genzyme) VS Parietex Composite (Sofradim) [48];<br>Sepramesh (Genzyme) VS PP mesh + polyurethane [48]	PP mesh + HA/CMC	Percentage of adhesion-area [48] rabbit);<br>Adhesion score composed of width, thickness, subjective strength and amount of adhesion [50](rabbit)
Rabbit	1 week	Sepramesh (Bard) VS Parietex composite (Covidien)	Polyester mesh + collagen	Rate of adhesion presence [49]
Rat<br>Rabbit	2 weeks	PP mesh + Seprafilm (Genzyme) VS Composix (Bard) [7];<br>Sepramesh (Bard) VS Proceed (Ethicon) [49];<br>Sepramesh (Genzyme) VS Marlex (Bard) + Vicryl [9]	No preference	Percentage of adhesion-area [7];<br>Percentage of adhesion-area [9];<br>Adhesion score composed of type and tenacity [9];<br>Rate of adhesion presence [49]
Rabbit	2 weeks	Sepramesh (Genzyme) VS Parietex Composite (Sofradim);<br>Sepramesh (Genzyme) VS PP mesh + polyurethane	PP mesh + HA/CMC	Percentage of mesh adhesion-area [48]
Rabbit	2 weeks	Sepramesh (Bard) VS Parietex Composite (Covidien)	Polyester mesh + collagen	Rate of adhesion presence [49]
Rat	3 weeks	Sepramesh (Genzyme) VS Parietex Composite (Sofradim)	Polyester mesh + collagen	Rate of adhesion presence [11];<br>Percentage of adhesion-area [11]
Rat	3 weeks	Sepramesh (Genzyme) VS Parietex Composite (Sofradim)	PP mesh + collagen	Rate of adhesion presence [11];<br>Percentage of adhesion-area [11]
Rat<br>Rabbit	3 weeks	Sepramesh (Genzyme) VS Bard Composix E/X (Davol) [11];<br>Sepramesh (Genzyme) VS Bard Composix (Bard) [51]	No preference	Rate of adhesion presence [11];<br>Extent score of adhesion [51]
Rat	3 weeks	Sepramesh (Genzyme) VS Bard Composix E/X (Davol) [11]	PP mesh + ePTFE	Percentage of adhesion-area [11]
Rat<br>Rabbit	4 weeks	Sepramesh (Genzyme) VS Parietex composite (Sofradim) [5];	PP mesh + HA/CMC	Percentage of adhesion-area [14] (rabbit), [5] (rat);<br>Breaking strength of graft-organ adhesions[18] (rat);

		Sepramesh (Genzyme) VS Vypro II (Johnson & Johnson) [18]; Sepramesh (Genzyme) VS Bard Composix mesh (Davol) [14]		Adhesion presence rate [14] (rabbit)
Rabbit	4 weeks	Sepramesh (Genzyme) VS Parietex Composite (Sofradim) [52]	Polyester mesh + collagen	Rate of bowel adhesion presence [52]; Adhesion-area [52]
Rat Rabbit Pig	4 weeks	Sepramesh (Genzyme) VS Parietex Composite (Sofradim) [4,52,18]; Sepramesh (Genzyme) VS Proceed (Ethicon) [4]; Sepramesh (Genzyme) VS PP mesh + Interceed (Johnson & Johnson) [17]; Sepramesh (Bard) VS Proceed (Ethicon) [53]; Sepramesh (Genzyme) VS Vypro II (Johnson & Johnson) [18]; PP mesh + Seprafilm (Genzyme) VS PP mesh + SurgiWrap (Mast Biosurgery) [54]; Sepramesh (Genzyme) VS Proceed (Ethicon)[55]; Sepramesh (Genzyme) VS PP mesh +Spray Gel [16]; Sepramesh (Bard) VS Ventrio Hernia Patch (Bard) [53]	No preference	Percentage of adhesion-area[4] (rat), [53] (pig), [52] (rabbit); Extent score of adhesion [17] (rat); Tenacity score of adhesion [53] (pig), [16,17] (rat); Breaking strength of graft-omentum adhesions [18] (rat); Breaking strength of graft-organ adhesions [18] (rat); Adhesion-area [54] (rat); Adhesion score composed of density and scale [55] (rat)
Rabbit	9 weeks	Sepramesh (Genzyme) VS Bard Composix (Bard)	No Preference	Extent score of adhesion [51]
Rat	3 months	Sepramesh (Genzyme) VS TiMesh (GfE Medizintechnik GmbH) [56]; Sepramesh (Genzyme) VS Vypro (Ethicon) [56]; Sepramesh (Genzyme) VS DynaMesh IPOM (FEG Textiltechnik) [56]; Sepramesh (Bard) VS Parietex Composite (Covidien) (M2) [24]; Sepramesh (Bard) VS Parietene Composite (Covidien) (M2,M3) [24]; Sepramesh (Bard) VS C-Qur Edge (Atrium) (M2) [24]; Sepramesh (Bard) VS Intramesh T1 (Cousin) (M2) [24]	No preference	Measurement1 (M1): Scale score of adhesion [56]; Measurement2 (M2): Percentage of adhesion-area [24]; Measurement3 (M3): Adhesion score composed of extent, type and tenacity [24]
Rat	3 months	Sepramesh (Bard) VS Parietex Composite (Covidien) (M3); Sepramesh (Bard) VS C-Qur Edge (M3); Sepramesh (Bard) VS Intramesh T1 (M3);	PP mesh + HA/CMC	Measurement2 (M2): Percentage of adhesion-area [24]; Measurement3 (M3): Adhesion score composed of extent, type and tenacity [24]
Rabbit	4 months	Sepramesh (Bard) VS Proceed (Ethicon)	PP mesh + HA/CMC	Tenacity score of adhesion [25]; Percentage of adhesion-area [25]
Rabbit	4 months	Sepramesh (Bard) VS Parietex Composite (Covidien) [25]; Sepramesh (Bard) VS Composix (Bard) [25]; Sepramesh (Genzyme) VS Composix (Bard) [51]; Sepramesh (Bard) VS C-Qur mesh (Atrium) [25]	No preference	Tenacity score of adhesion [25]; Percentage of adhesion-area [25]; Adhesion score of extent [51]
Rabbit	5 months	Sepramesh (Genzyme) VS Parietex Composite (Sofradim)	No preference	Percentage of adhesion-area [52]
Rabbit	5 months	Sepramesh (Genzyme) VS Parietex Composite (Sofradim)	Polyester mesh + collagen	Rate of bowel adhesion presence [52]; Adhesion-area [52]

Table 13 The comparison between polyester mesh + collagen (Parietex composite mesh) and other meshes with coatings

Species	Follow-up	Comparisons and reference	Best mesh	Measurements and reference
Rabbit	3 days	Parietex Composite (Sofradim) VS Sepramesh (Genzyme) [48]; Parietex Composite (Covidien) VS Sepramesh (Bard) [49]; Parietex Composite (Covidien) VS Proceed (Ethicon) [57,49]	Polyester mesh + collagen	Percentage of mesh adhesion-area [48,57]; Rate of adhesion presence [49]
Rabbit	3 days	Parietex Composite (Sofradim) VS PP mesh + PU [48]; Parietex Composite (Covidien) VS Ventralex ST Hernia Patch (Bard) [57]	No preference	Percentage of mesh adhesion-area [48,57]
Rabbit Rat	7 days	Parietex Composite (Covidien) VS Proceed (Ethicon)[57,50,49]; Parietex Composite (Sofradim) VS Proceed (Ethicon) [4]; Parietex Composite (Sofradim) VS Ultrapro (Ethicon) [4]; Parietex Composite (Sofradim) VS Timesh (GfE) [4]; Parietex Composite (Sofradim) VS Sepramesh (Genzyme) [48]; Parietex Composite (Covidien) VS Sepramesh (Bard) [49]	Polyester mesh + collagen	Percentage of adhesion-area [4,57]; Rate of adhesion presence [49]; Percentage of mesh adhesion-area [48]; Adhesion score composed of width, thickness, subjective strength and amount of adhesion [50]
Rabbit Rat	7 days	Parietex Composite (Covidien) VS Ventralex ST Hernia Patch (Bard) [57]; Parietex Composite (Sofradim) VS Sepramesh (Genzyme) [4]; Parietex Composite (Covidien) VS Bard Sepramesh Composite (Bard) [50]; Parietex Composite (Sofradim) VS Sepramesh (Genzyme) [5]; Parietex Composite (Sofradim) VS Timesh (GfE Medizintechnik) [26]; Parietex Composite (Sofradim) VS Proceed (Ethicon) [58,50,26]; Parietex Composite (Sofradim) VS Ultrapro (Ethicon) [26]; Parietex Composite (Sofradim) VS C-Qur (Atrium) [26]; Parietex Composite (Sofradim) VS PP mesh + PU [48]; Parietex Composite (Sofradim) VS PP mesh + polylactide composite [58]	No preference	Percentage of adhesion-area [48,4,57,26,5]; Numbers of rats with bowel adhesion [5]; Adhesion score composed of extent, type and tenacity [26]; Adhesion score composed of width, thickness, subjective strength and amount of adhesion [50]; Extent score of adhesion [58]
Rat	10 days	Parietex Composite VS PP mesh + Polydopamine	No preference	Adhesion score composed of type, intensity and area [59]
Rabbit	2 weeks	Proceed Ventral Patch (Ethicon) VS Ventralex ST Hernia Patch (Bard) [60,57]; Parietex Composite (Sofradim) VS Composix (Bard) [27]; Parietex Composite (Sofradim) VS Prolene (Ethicon) + ePTFE (Gore-Tex) [28]; Parietex Composite (Sofradim) VS PP mesh + polyurethane [48,27]	No preference	Percentage of adhesion-area [48,60,57,27]; Adhesion-area [28]

Rabbit<br>Pig	2 weeks	Parietex Composite (Sofradim) VS Sepramesh (Genzyme) [48];<br>Parietex Composite (Covidien) VS Sepramesh (Bard) [49];<br>Parietex Composite(Sofradim) VS Vypro (Ethicon) [61];<br>Parietex Composite Ventral Patch (Covidien) VS Proceed Ventral Patch (Ethicon) [60,57];<br>Parietex Composite (Sofradim) VS Composix E/X (Bard) [62]	Polyester mesh +collagen	Percentage of mesh adhesion-area [48,62];<br>Rate of adhesion presence [49];<br>Adhesion-area [61];<br>Percentage of adhesion-area [60,57];<br>Density score of adhesion [62]
Rat	3 weeks	Parietex Composite (Sofradim) VS Sepramesh (Genzyme)	Polyester mesh + collagen	Rate of adhesion presence [11];<br>Percentage of adhesion-area [11]
Rat	3 weeks	Parietex Composite (Sofradim) VS Parietene Composite (Sofradim);<br>Parietex Composite (Sofradim) VS Dual Mesh (Gore-Tex)	No preference	Rate of adhesion presence [11];<br>Percentage of adhesion-area [11]
Rat	4 weeks	Parietex composite (Sofradim) VS Sepramesh (Genzyme)	PP mesh + HA/CMC	Percentage of adhesion-area [5]
Pig	4 weeks	Parietex Composite (Sofradim) VS Proceed (Ethicon)	PP mesh + ORC	Percentage of adhesion-area [63]
Rat<br>Rabbit	4 weeks	Parietex Composite (Covidien) VS Ultrapro (Ethicon) [4];<br>Parietex Composite (Covidien) VS Timesh (GfE) [4];<br>Parietex Composite (Covidien) VS Proceed (Ethicon) [4,26];<br>Parietex composite (Sofradim) VS Vypro II (Johnson & Johnson) [18];<br>Parietex composite (Sofradim) VS Sepramesh (Genzyme) [52];<br>Parietex Composite (Covidie) VS PP mesh + polylactide composite[58]	Polyester mesh + collagen	Percentage of adhesion-area [4] (rat);<br>Breaking strength of graft–organ [18] (rat);<br>Rate of bowel adhesion presence [52] (rabbit);<br>Adhesion-area [52] (rabbit);<br>Adhesion score composed of extent, type and tenacity [26] (rat)
Rat<br>Rabbit<br>Pig	4 weeks	Parietex Composite (Sofradim) VS Sepramesh (Genzyme) [52,18,4];<br>Parietex Composite (Sofradim) VS Vypro II (Johnson & Johnson) [18];<br>Parietex Composite (Sofradim) VS Timesh (GfE) [26];<br>Parietex Composite (Sofradim) VS Proceed (Ethicon) [26];<br>Parietex Composite (Sofradim) VS Ultrapro (Ethicon) [26];<br>Parietex Composite (Sofradim) VS C-Qur (Atrium) [26];<br>Parietex Composite (Sofradim) VS Composix E/X (Bard) [63];<br>Parietex Composite (Covidien) VS Proceed (Ethicon) [58];<br>Parietex Composite (Covidien) VS PP mesh + polyglycolic acid [64]	No preference	Percentage of adhesion-area [4] (rat), [63] (pig), [52] (rabbit), [26] (rat);<br>Breaking strength of graft–omentum [18] (rat);<br>Extent score of adhesion [58] (rabbit);<br>Number of bands of adhesions between viscera and abdominal wall[64] (rabbit)
Rabbit	6 weeks	Parietex Composite Ventral Patch (Covidien) VS Proceed Ventral Patch	Polyester mesh + collagen	Percentage of adhesion-area([60,57]
Rabbit	6 weeks	Parietex Composite Ventral Patch (Covidien) VS Ventralex ST Hernia Patch (Bard)	No preference	Percentage of adhesion-area [60,57]
Rat	2 months	Parietex Composite VS PP mesh + polydopamine	No preference	Adhesion score composed of type, intensity and area [59]
Pig<br>Rabbit	3 months	Parietex Composite VS PP mesh + polyglycolic acid[64]<br>Parietex Composite (Covidien) VS Parietene Composite	No preference	Number of bands of adhesions between viscera and abdominal wall [64] (rabbit);

Rat		(Covidien) (M2,M3) [24];<br>Parietex Composite (Covidien) VS C-Qur Edge (Atrium) (M2,M3) [24];<br>Parietex Composite (Covidien) VS Sepramesh IP (Bard) (M3) [24];<br>Parietex Composite (Covidien) VS Intramesh T1 (Cousin) (M2,M3) [24]		Measurement 2 (M2): Percentage of adhesion-area[24](rat);<br>Measurement 3 (M3): Adhesion score composed of extent, type and tenacity [24] (rat)
Sheep	3 months	Parietex Composite (Covidien) VS DynaMesh-IPOM (Kebo)	Polyester mesh + collagen	Extent score of adhesion [65];<br>Tenacity score of adhesion [65]
Rat	3 months	Parietex Composite (Covidien) VS Sepramesh IP (Bard)	PP mesh + HA/CMC	Percentage of adhesion-area [24]
Rabbit	4 months	Parietex Composite (Covidien) VS Proceed (Ethicon) [58];<br>Parietex Composite (Covidien) VS PP mesh + polylactide composite [58];<br>Parietex Composite (Covidien) VS Proceed (Ethicon) [25];<br>Parietex Composite (Covidien) VS Sepramesh (Bard) [25];<br>Parietex Composite (Covidien) VS Composix (Bard) [25];<br>Parietex Composite (Covidien) VS C-Qur mesh (Atrium) [25]	No preference	Extent score of adhesion [58];<br>Tenacity score of adhesion [58];<br>Adhesion score of tenacity and type [25];<br>Percentage of adhesion-area [25]
Rabbit	5 months	Parietex composite (Sofradim) VS Sepramesh (Genzyme)	Polyester mesh + collagen	Rate of bowel adhesion presence [52];<br>Adhesion-area [52]
Rabbit	5 months	Parietex composite (Sofradim) VS Sepramesh (Genzyme)	No preference	Percentage of adhesion-area [52]
Sheep<br>Rabbit	6 months	Parietex Composite (Covidien) VS DynaMesh-IPOM (Kebo) [65];<br>Parietex Composite Ventral Patch (Covidien) VS Proceed (Ethicon) [57]	Polyester mesh + collagen	Extent score of adhesion [65];<br>Tenacity score of adhesion [65];<br>Percentage of adhesion-area [57]
Rabbit	6 months	Parietex Composite Ventral Patch (Covidien) VS Ventralex ST Hernia Patch (Bard)	No preference	Percentage of adhesion-area [57]
Sheep	12 months	Parietex Composite (Covidien) VS DynaMesh-IPOM (Kebo)	Polyester mesh + collagen	Extent score of adhesion [65];<br>Tenacity score of adhesion [65]
Sheep	18 months	Parietex Composite (Covidien) VS DynaMesh-IPOM (Kebo)	Polyester mesh + collagen	Extent score of adhesion [65];<br>Tenacity score of adhesion [65]

References

1. Alponat A, Lakshminarasappa SR, Teh M, Rajnakova A, Moochhala S, Goh PM, Chan ST (1997) Effects of physical barriers in prevention of adhesions: an incisional hernia model in rats. The Journal of surgical research 68 (2):126-132. doi:10.1006/jsre.1996.4979

2. Felemovicius I, Bonsack ME, Hagerman G, Delaney JP (2004) Prevention of adhesions to polypropylene mesh. Journal of the American College of Surgeons 198 (4):543-548. doi:10.1016/j.jamcollsurg.2003.12.004

3. Malazgirt Z, Ulusoy AN, Gok Y, Karagoz F, Tac K (2000) Bioabsorbable membrane prevents adhesions to polypropylene mesh in rats. Hernia : the journal of hernias and abdominal wall surgery 4 (3):129-133

4. Burger JW, Halm JA, Wijsmuller AR, ten Raa S, Jeekel J (2006) Evaluation of new prosthetic meshes for ventral hernia repair. Surgical endoscopy 20 (8):1320-1325. doi:10.1007/s00464-005-0706-4

5. van 't Riet M, de Vos van Steenwijk PJ, Bonthuis F, Marquet RL, Steyerberg EW, Jeekel J, Bonjer HJ (2003) Prevention of adhesion to prosthetic mesh: comparison of different barriers using an incisional hernia model. Annals of surgery 237 (1):123-128. doi:10.1097/01.sla.0000041264.79870.fc

6. Baptista ML, Bonsack ME, Delaney JP (2000) Seprafilm reduces adhesions to polypropylene mesh. Surgery 128 (1):86-92. doi:10.1067/msy.2000.106810

7. Besim H, Yalcin Y, Hamamci O, Arslan K, Sonisik M, Korkmaz A, Erdogan S (2002) Prevention of intraabdominal adhesions produced by polypropylene mesh. European surgical research Europaische chirurgische Forschung Recherches chirurgicales europeennes 34 (3):239-243. doi:10.1159/000063395

8. Ozmen MM, Aslar AK, Terzi MC, Albayrak L, Berberoglu M (2002) Prevention of adhesions by bioresorbable tissue barrier following laparoscopic intraabdominal mesh insertion. Surgical Laparoscopy, Endoscopy and Percutaneous Techniques 12 (5):342-346

9. Bellon JM, Garcia-Honduvilla N, Serrano N, Rodriguez M, Pascual G, Bujan J (2005) Composite prostheses for the repair of abdominal wall defects: effect of the structure of the adhesion barrier

component. Hernia : the journal of hernias and abdominal wall surgery 9 (4):338-343. doi:10.1007/s10029-005-0012-z

10. Szabo A, Haj M, Waxsman I, Eitan A (2000) Evaluation of seprafilm and amniotic membrane as adhesion prophylaxis in mesh repair of abdominal wall hernia in rats. European surgical research Europaische chirurgische Forschung Recherches chirurgicales europeennes 32 (2):125-128. doi:10.1159/000008751

11. Gonzalez R, Rodeheaver GT, Moody DL, Foresman PA, Ramshaw BJ (2004) Resistance to adhesion formation: a comparative study of treated and untreated mesh products placed in the abdominal cavity. Hernia : the journal of hernias and abdominal wall surgery 8 (3):213-219. doi:10.1007/s10029-004-0213-x

12. Borrazzo EC, Belmont MF, Boffa D, Fowler DL (2004) Effect of prosthetic material on adhesion formation after laparoscopic ventral hernia repair in a porcine model. Hernia : the journal of hernias and abdominal wall surgery 8 (2):108-112. doi:10.1007/s10029-003-0181-6

13. Mayagoitia-Gonzalez JC, Gudino-Amezcua LM, Rivera-Barragan V, Mellado-Diaz AV, Diaz-Chavez EP (2012) Prevention of intestinal adhesions as a result of intraperitoneal mesh with the addition of hyaluronic acid/carboxymethylcellulose gel. Experimental model in rats. Cir Cir 80 (2):150-156

14. Greenawalt KE, Butler TJ, Rowe EA, Finneral AC, Garlick DS, Burns JW (2000) Evaluation of sepramesh biosurgical composite in a rabbit hernia repair model. The Journal of surgical research 94 (2):92-98. doi:10.1006/jsre.2000.6020

15. Dinsmore RC, Calton WC, Jr., Harvey SB, Blaney MW (2000) Prevention of adhesions to polypropylene mesh in a traumatized bowel model. Journal of the American College of Surgeons 191 (2):131-136

16. Dasiran F, Eryilmaz R, Isik A, Okan I, Somay A, Sahin M (2015) The effect of polyethylene glycol adhesion barrier (Spray Gel) on preventing peritoneal adhesions. Bratislavske lekarske listy 116 (6):379-382

17. Dilege E, Coskun H, Gunduz B, Sakiz D, Mihmanli M (2006) Prevention of adhesion to prosthetic mesh in incisional ventral hernias: comparison of different barriers in an experimental model. European surgical research Europaische chirurgische Forschung Recherches chirurgicales europeennes 38 (3):358-364. doi:10.1159/000094382

18. Kayaoglu HA, Ozkan N, Hazinedaroglu SM, Ersoy OF, Erkek AB, Koseoglu RD (2005) Comparison of adhesive properties of five different prosthetic materials used in hernioplasty. Journal of investigative surgery : the official journal of the Academy of Surgical Research 18 (2):89-95. doi:10.1080/08941930590926357

19. Yelimlies B, Alponat A, Cubukcu A, Kuru M, Oz S, Ercin C, Gonullu N (2003) Carboxymethylcellulose coated on visceral face of polypropylene mesh prevents adhesion without impairing wound healing in incisional hernia model in rats. Hernia : the journal of hernias and abdominal wall surgery 7 (3):130-133. doi:10.1007/s10029-003-0125-1

20. Kramer K, Senninger N, Herbst H, Probst W (2002) Effective prevention of adhesions with hyaluronate. Archives of surgery (Chicago, Ill : 1960) 137 (3):278-282

21. Erpek H, Tuncyurek P, Soyder A, Boylu S (2006) Hyaluronic acid/carboxymethylcellulose membrane barrier versus taurolidine for the prevention of adhesions to polypropylene mesh. European surgical research Europaische chirurgische Forschung Recherches chirurgicales europeennes 38 (4):414-417. doi:10.1159/000094748

22. Sikkink CJ, Vries de Reilingh TS, Malyar AW, Jansen JA, Bleichrodt RP, van Goor H (2006) Adhesion formation and reherniation differ between meshes used for abdominal wall reconstruction. Hernia : the journal of hernias and abdominal wall surgery 10 (3):218-222. doi:10.1007/s10029-006-0065-7

23. Nohuz E, Alaboud M, Darcha C, Alloui A, Aublet-Cuvelier B, Jacquetin B (2014) Effectiveness of Hyalobarrier and Seprafilm to prevent polypropylene mesh shrinkage: a macroscopic and histological experimental study. International urogynecology journal 25 (8):1081-1087. doi:10.1007/s00192-014-2357-2

24. Schreinemacher MH, van Barneveld KW, Dikmans RE, Gijbels MJ, Greve JW, Bouvy ND (2013) Coated meshes for hernia repair provide comparable intraperitoneal adhesion prevention. Surgical endoscopy 27 (11):4202-4209. doi:10.1007/s00464-013-3021-5

25. Pierce RA, Perrone JM, Nimeri A, Sexton JA, Walcutt J, Frisella MM, Matthews BD (2009) 120-day comparative analysis of adhesion grade and quantity, mesh contraction, and tissue response to a novel omega-3 fatty acid bioabsorbable barrier macroporous mesh after intraperitoneal placement. Surgical innovation 16 (1):46-54. doi:10.1177/1553350608330479

26. Schreinemacher MH, Emans PJ, Gijbels MJ, Greve JW, Beets GL, Bouvy ND (2009) Degradation of mesh coatings and intraperitoneal adhesion formation in an experimental model. The British journal of surgery 96 (3):305-313

27. Bellon JM, Rodriguez M, Garcia-Honduvilla N, Pascual G, Gil VG, Bujan J (2007) Peritoneal effects of prosthetic meshes used to repair abdominal wall defects: monitoring adhesions by sequential laparoscopy. Journal of Laparoendoscopic and Advanced Surgical Techniques 17 (2):160-166

28. Bellon JM, Garcia-Carranza A, Jurado F, Garcia-Honduvilla N, Carrera-San Martin A, Bujan J (2001) Peritoneal regeneration after implant of a composite prosthesis in the abdominal wall. World journal of surgery 25 (2):147-152

29. Lamber B, Grossi JV, Manna BB, Montes JH, Bigolin AV, Cavazzola LT (2013) May polyester with collagen coating mesh decrease the rate of intraperitoneal adhesions in incisional hernia repair? Arquivos brasileiros de cirurgia digestiva : ABCD = Brazilian archives of digestive surgery 26 (1):13-17

30. Biondo-Simoes ML, Carvalho LB, Conceicao LT, Santos KB, Schiel WA, Arantes M, Silveira TD, Magri JC, Gomes FF (2017) Comparative study of Polypropylene versus Parietex composite(R), Vicryl(R) and Ultrapro(R) meshes, regarding the formation of intraperitoneal adhesions. Acta cirurgica brasileira 32 (2):98-107. doi:10.1590/s0102-865020170202

31. Voskerician G, Jin J, White MF, Williams CP, Rosen MJ (2010) Effect of biomaterial design criteria on the performance of surgical meshes for abdominal hernia repair: a pre-clinical evaluation in a

chronic rat model. Journal of materials science Materials in medicine 21 (6):1989-1995. doi:10.1007/s10856-010-4037-1

32. Ditzel M, Deerenberg EB, Grotenhuis N, Harlaar JJ, Monkhorst K, Bastiaansen-Jenniskens YM, Jeekel J, Lange JF (2013) Biologic meshes are not superior to synthetic meshes in ventral hernia repair: an experimental study with long-term follow-up evaluation. Surgical endoscopy 27 (10):3654-3662. doi:10.1007/s00464-013-2939-y

33. van't Riet M, Burger JW, Bonthuis F, Jeekel J, Bonjer HJ (2004) Prevention of adhesion formation to polypropylene mesh by collagen coating: a randomized controlled study in a rat model of ventral hernia repair. Surgical endoscopy 18 (4):681-685. doi:10.1007/s00464-003-9054-4

34. Aliabadi-Wahle S, Choe EU, Jacob-LaBarre J, Flint LM, Ferrara JJ (1996) Evaluation of a novel synthetic material for closure of large abdominal wall defects. Surgery 119 (2):141-145

35. Bellon JM, Garcia-Carranza A, Jurado F, Garcia-Honduvilla N, Carrera-San Martin A, Bujan J (2002) Evaluation of a new composite prosthesis (PL-PU99) for the repair of abdominal wall defects in terms of behavior at the peritoneal interface. World journal of surgery 26 (6):661-666

36. Ansaloni L, Catena F, Coccolini F, Fini M, Gazzotti F, Giardino R, Pinna AD (2009) Peritoneal adhesions to prosthetic materials: an experimental comparative study of treated and untreated polypropylene meshes placed in the abdominal cavity. Journal of laparoendoscopic & advanced surgical techniques Part A 19 (3):369-374. doi:10.1089/lap.2008.0366

37. Emans PJ, Schreinemacher MH, Gijbels MJ, Beets GL, Greve JW, Koole LH, Bouvy ND (2009) Polypropylene meshes to prevent abdominal herniation. Can stable coatings prevent adhesions in the long term? Annals of biomedical engineering 37 (2):410-418. doi:10.1007/s10439-008-9608-7

38. Demir U, Mihmanli M, Coskun H, Dilege E, Kalyoncu A, Altinli E, Gunduz B, Yilmaz B (2005) Comparison of prosthetic materials in incisional hernia repair. Surgery today 35 (3):223-227. doi:10.1007/s00595-004-2907-1

39. Fitzgibbons Jr RJ, Salerno GM, Filipi CJ, Hunter WJ, Watson P (1994) A laparoscopic intraperitoneal onlay mesh technique for the repair of an indirect inguinal hernia. Annals of surgery 219 (2):144-156
40. Aramayo AL, Lopes Filho Gde J, Barbosa Cde A, Amaral Vda F, Costa LA (2013) Abdominal wall healing in incisional hernia using different biomaterials in rabbits. Acta cirurgica brasileira 28 (4):307-316
41. Rossi LF, Trindade MRM, AJ DA, Meurer L (2017) PERITONEAL ADHESIONS TYPE I, III AND TOTAL COLLAGEN ON POLYPROPYLENE AND COATED POLYPROPYLENE MESHES: EXPERIMENTAL STUDY IN RATS. Arquivos brasileiros de cirurgia digestiva : ABCD = Brazilian archives of digestive surgery 30 (2):77-82. doi:10.1590/0102-6720201700020001
42. Novitsky YW, Harrell AG, Cristiano JA, Paton BL, Norton HJ, Peindl RD, Kercher KW, Heniford BT (2007) Comparative evaluation of adhesion formation, strength of ingrowth, and textile properties of prosthetic meshes after long-term intra-abdominal implantation in a rabbit. The Journal of surgical research 140 (1):6-11. doi:10.1016/j.jss.2006.09.015
43. Bellon JM, Rodriguez M, Garcia-Honduvilla N, Gomez-Gil V, Pascual G, Bujan J (2008) Postimplant behavior of lightweight polypropylene meshes in an experimental model of abdominal hernia. Journal of Investigative Surgery 21 (5):280-287
44. de Vries Reilingh TS, van Goor H, Koppe MJ, Bodegom ME, Hendriks T, Bleichrodt RP (2007) Interposition of Polyglactin Mesh Does Not Prevent Adhesion Formation Between Viscera and Polypropylene Mesh. Journal of Surgical Research 140 (1):27-30
45. Goldenberg A, Matone J, Marcondes W, Herbella FA, Farah JF (2005) Comparative study of inflammatory response and adhesions formation after fixation of different meshes for inguinal hernia repair in rabbits. Acta cirurgica brasileira / Sociedade Brasileira para Desenvolvimento Pesquisa em Cirurgia 20 (5):347-352
46. Liu L, Petro C, Majumder A, Fayezizadeh M, Anderson J, Novitsky YW (2016) The use of Vicryl mesh in a porcine model to assess its safety as an adjunct to posterior fascial closure during

retromuscular mesh placement. Hernia : the journal of hernias and abdominal wall surgery 20 (2):289-295. doi:10.1007/s10029-016-1469-7

47. Schug-Pass C, Sommerer F, Tannapfel A, Lippert H, Kockerling F (2009) The use of composite meshes in laparoscopic repair of abdominal wall hernias: are there differences in biocompatibily?: experimental results obtained in a laparoscopic porcine model. Surgical endoscopy 23 (3):487-495. doi:10.1007/s00464-008-0085-8

48. Bellon JM, Rodriguez M, Garcia-Honduvilla N, Gomez-Gil V, Pascual G, Bujan J (2007) Real-time monitoring of the peritoneal behavior of composite prostheses by sequential laparoscopy: Applicability in ventral hernia repair. [Spanish] (Seguimiento del comportamiento peritoneal de diferentes biomateriales empleando laparoscopia secuencial. Aplicabilidad en la reparacion de hernias ventrales.). Cirugia espanola 82 (5):290-296

49. Rodriguez M, Pascual G, Sotomayor S, Perez-Koihler B, Cifuentes A, Bellon JM (2011) Chemical adhesion barriers: Do they affect the intraperitoneal behavior of a composite mesh? Journal of Investigative Surgery 24 (3):115-122

50. Konerding MA, Chantereau P, Delventhal V, Holste JL, Ackermann M (2012) Biomechanical and histological evaluation of abdominal wall compliance with intraperitoneal onlay mesh implants in rabbits: a comparison of six different state-of-the-art meshes. Medical engineering & physics 34 (7):806-816. doi:10.1016/j.medengphy.2011.09.022

51. Matthews BD, Mostafa G, Carbonell AM, Joels CS, Kercher KW, Austin C, Norton HJ, Heniford BT (2005) Evaluation of adhesion formation and host tissue response to intra-abdominal polytetrafluoroethylene mesh and composite prosthetic mesh. Journal of Surgical Research 123 (2):227-234

52. Johnson EK, Hoyt CH, Dinsmore RC (2004) Abdominal wall hernia repair: a long-term comparison of Sepramesh and Dualmesh in a rabbit hernia model. The American surgeon 70 (8):657-661

53. Deeken CR, Matthews BD (2011) Comparison of contracture, adhesion, tissue ingrowth, and histologic response characteristics of permanent and absorbable barrier meshes in a porcine model of laparoscopic ventral hernia repair. Hernia : the journal of hernias and abdominal wall surgery:1-8

54. Gruber-Blum S, Petter-Puchner AH, Brand J, Fortelny RH, Walder N, Oehlinger W, Koenig F, Redl H (2011) Comparison of three separate antiadhesive barriers for intraperitoneal onlay mesh hernia repair in an experimental model. The British journal of surgery 98 (3):442-449. doi:10.1002/bjs.7334

55. Leblebici IM, Bozkurt S, Sever B (2014) Comparison of adhesion-preventing meshes in a rat model of incisional hernia in terms of intra-abdominal adhesions and other complications. Biomedical Research (India) 25 (3):414-419

56. Celik A, Altinli E, Koksal N, Celik AS, Onur E, Ozkan OF, Gumrukcu G (2009) The shrinking rates of different meshes placed intraperitoneally: a long-term comparison of the TiMesh, VYPRO II, Sepramesh, and DynaMesh. Surgical laparoscopy, endoscopy & percutaneous techniques 19 (4):e130-134. doi:10.1097/SLE.0b013e3181aa598d

57. Garcia-Moreno F, Sotomayor S, Perez-Lopez P, Perez-Kohler B, Bayon Y, Pascual G, Bellon JM (2014) Intraperitoneal behaviour of a new composite mesh (Parietex Composite Ventral Patch) designed for umbilical or epigastric hernia repair. Surgical endoscopy 28 (12):3479-3488. doi:10.1007/s00464-014-3633-4

58. Dolce CJ, Keller JE, Stefanidis D, Walters KC, Heath JJ, Lincourt AL, Norton HJ, Kercher KW, Heniford BT (2012) Evaluation of soft tissue attachments to a novel intra-abdominal prosthetic in a rabbit model. Surgical innovation 19 (3):295-300. doi:10.1177/1553350611429115

59. Hu M, Lin X, Huang R, Yang K, Liang Y, Zhang X, Wang H, Wu D (2018) Lightweight, Highly Permeable, Biocompatible, and Antiadhesive Composite Meshes for Intraperitoneal Repairs. Macromolecular bioscience 18 (7):e1800067. doi:10.1002/mabi.201800067

60. Garcia-Moreno F, Perez-Lopez P, Sotomayor S, Perez-Kohler B, Bayon Y, Pascual G, Bellon JM (2015) Comparing the host tissue response and peritoneal behavior of composite meshes used for ventral hernia repair. The Journal of surgical research 193 (1):470-482. doi:10.1016/j.jss.2014.07.049

61. Bellon JM, Garcia-Honduvilla N, Jurado F, Garcia-Carranza A, Garcia-Moreno F, Martin AC, Bujan J (2001) Use of composite prostheses in the repair of defects in the abdominal wall: prosthetic behaviour at the peritoneum. The European journal of surgery = Acta chirurgica 167 (9):666-671. doi:10.1080/11024150152619291
62. Duffy AJ, Hogle NJ, LaPerle KM, Fowler DL (2004) Comparison of two composite meshes using two fixation devices in a porcine laparoscopic ventral hernia repair model. Hernia : the journal of hernias and abdominal wall surgery 8 (4):358-364. doi:10.1007/s10029-004-0258-x
63. Jacob BP, Hogle NJ, Durak E, Kim T, Fowler DL (2007) Tissue ingrowth and bowel adhesion formation in an animal comparative study: polypropylene versus Proceed versus Parietex Composite. Surgical endoscopy 21 (4):629-633. doi:10.1007/s00464-006-9157-9
64. de Araujo UR, Czeczko NG, Ribas-Filho JM, Malafaia O, Budel VM, Balderrama CM, Zimmermann E, Dietz UA (2009) Intraperitoneal meshes in the repair of abdominal wall defects: comparison of polyester with collagen versus polypropylene with polyglycolic acid. Revista do Colegio Brasileiro de Cirurgioes 36 (3):241-249
65. Zinther NB, Wara P, Friis-Andersen H (2010) Intraperitoneal onlay mesh: an experimental study of adhesion formation in a sheep model. Hernia : the journal of hernias and abdominal wall surgery 14 (3):283-289. doi:10.1007/s10029-009-0622-y
